# Integrative multiomics analysis identifies molecular subtypes and potential targets of hepatocellular carcinoma

**DOI:** 10.1002/ctm2.1727

**Published:** 2024-05-28

**Authors:** Shuai Yang, Lu Zheng, Lingling Li, Jiangang Zhang, Jingchun Wang, Huakan Zhao, Yu Chen, Xudong Liu, Hui Gan, Junying Chen, Mei Yan, Chuanyin He, Kai Li, Chen Ding, Yongsheng Li

**Affiliations:** ^1^ Department of Hematology & Oncology Jiangbei Campus The First Affiliated Hospital of Army Medical University Chongqing China; ^2^ Department of Hepatobiliary Surgery Xinqiao Hospital Army Medical University Chongqing China; ^3^ State Key Laboratory of Genetic Engineering Institutes of Biomedical Sciences Human Phenome Institute School of Life Sciences Zhongshan Hospital Fudan University Shanghai China; ^4^ Department of Medical Oncology Chongqing University Cancer Hospital Chongqing China; ^5^ Department of Gastroenterology Xinqiao Hospital, Army Medical University Chongqing China; ^6^ Department of Radiology Xinqiao Hospital Army Medical University Chongqing China; ^7^ Department of Pathology Jiangbei Campus The First Affiliated Hospital of Army Medical University Chongqing China

**Keywords:** exportin 1, 5‐lipoxygenase, molecular subtypes, neutrophil degranulation, ribosome biogenesis

## Abstract

**Background:**

The liver is anatomically divided into eight segments based on the distribution of Glisson's triad. However, the molecular mechanisms underlying each segment and its association with hepatocellular carcinoma (HCC) heterogeneity are not well understood. In this study, our objective is to conduct a comprehensive multiomics profiling of the segmentation atlas in order to investigate potential subtypes and therapeutic approaches for HCC.

**Methods:**

A high throughput liquid chromatography‐tandem mass spectrometer strategy was employed to comprehensively analyse proteome, lipidome and metabolome data, with a focus on segment‐resolved multiomics profiling. To classify HCC subtypes, the obtained data with normal reference profiling were integrated. Additionally, potential therapeutic targets for HCC were identified using immunohistochemistry assays. The effectiveness of these targets were further validated through patient‐derived organoid (PDO) assays.

**Results:**

A multiomics profiling of 8536 high‐confidence proteins, 1029 polar metabolites and 3381 nonredundant lipids was performed to analyse the segmentation atlas of HCC. The analysis of the data revealed that in normal adjacent tissues, the left lobe was primarily involved in energy metabolism, while the right lobe was associated with small molecule metabolism. Based on the normal reference atlas, HCC patients with segment‐resolved classification were divided into three subtypes. The C1 subtype showed enrichment in ribosome biogenesis, the C2 subtype exhibited an intermediate phenotype, while the C3 subtype was closely associated with neutrophil degranulation. Furthermore, using the PDO assay, exportin 1 (XPO1) and 5‐lipoxygenase (ALOX5) were identified as potential targets for the C1 and C3 subtypes, respectively.

**Conclusion:**

Our extensive analysis of the segmentation atlas in multiomics profiling defines molecular subtypes of HCC and uncovers potential therapeutic strategies that have the potential to enhance the prognosis of HCC.

## Graphical abstract

- Integrative multiomics profiling was performed to create a segmentation atlas of liver tissues and HCC.
- The metabolism in the normal adjacent tissues of the left and right lobes exhibited differential characteristics.
- HCC was classified into 3 subtypes based on the segmentation atlas, namely S‐RB, S‐Im and S‐ND.
- Further analysis using PDO assays revealed that XPO1 and ALOX5 could be potential targets for the S‐RB and S‐NB subtypes, respectively.

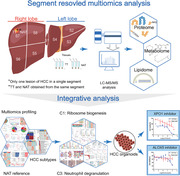

## 1 INTRODUCTION

**FIGURE 1:**
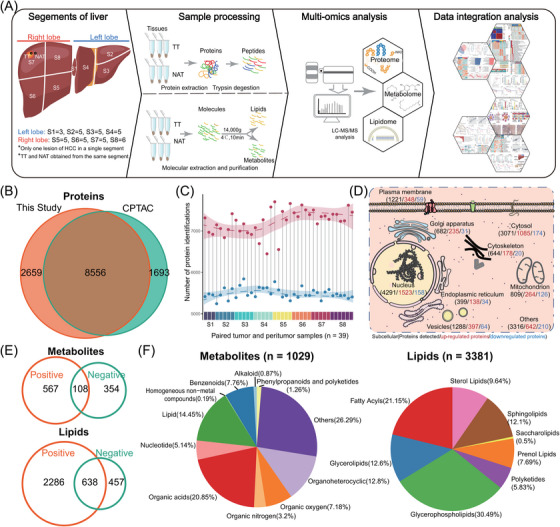
Multiomics characterisation of early‐stage HCC samples. (A) Schematic summary of experiments and integrated Multiomics analyses, including liver segmentation, untargeted LC‐MS/MS data generation and integration analysis. (B) Venn diagram depicts the overlap proteins of this study and CPTAC HCC cohort. (C) Overview of the protein identifications in segment‐resolved HCC samples. The dashed curves fitted by lasso regression represent the distribution of protein identifications. The shading that underlies the lasso curves denotes the 95% confidence intervals. (D) Subcellular distribution of identified proteins investigated by the human protein atlas. (E) Venn diagrams show the overlap metabolites and lipids identified in positive‐ and negative‐ion mode of LC‐MS/MS. (F) Pie charts indicate the categories of identified metabolites and lipids annotated by Human Metabolome Database (HMDB). CPTAC, Clinical Proteomic Tumor Analysis Consortium; HCC, hepatocellular carcinoma; LC‐MS/MS, liquid chromatography‐tandem mass spectrometer.

Hepatocellular carcinoma (HCC) is the most common type of liver cancer and is characterised by its rapid progression, resulting in a low 5‐year survival rate of only 18%.^1^ Despite significant advancements in HCC treatments, such as anti‐angiogenic targeted therapy and immunotherapy, the response rates for these promising treatments remain below 30%.^2^ The liver is anatomically divided into eight functional segments that are centred on the Glisson system. These segments are determined by the portal vein, hepatic artery and bile drainage.^3^ The hepatic segments, although histologically identical, may have variations in their microenvironment due to the different branches of the Glisson system originating from the alimentary canal. The liver is composed of the left lobe (segments 2−4, S2–S4) and the right lobe (segments 5−8, S5–S8). Additionally, there is a distinct segment called the caudate lobe (S1), which is located adjacent to the ligamentum venosum and the inferior vena cava (Figure 1A). Therefore, conducting a comprehensive analysis of multiomics is clinically important to uncover the unique characteristics of each liver segment or lobe. This understanding will contribute to a better comprehension of the heterogeneity of HCC originating from different hepatic segments.

As HCC is highly heterogeneous, preliminary studies involving proteomic and genomic characterisation can be valuable in understanding the different molecular subtypes and providing personalised treatment options.^4^, ^5^, ^6^, ^7^ Transcriptomics and proteomics provide insights into gene and protein expression, while metabolomics offers a functional readout of metabolic processes, allowing for a direct assessment of molecular phenotype and pathophysiological conditions.^8^ Dysregulated metabolism is a hallmark of cancer, including HCC.^9^ The impaired degradation of lipid alterations is known to contribute to hepatocarcinogenesis.^10^ However, the lack of high throughput metabolomics data has hindered the integration and analysis of HCC from multiomics data, including the metabolome or lipidome. Here, we aimed to create a comprehensive segment‐resolved atlas of the proteome, metabolome and lipidome in tumour tissues (TT) and normal adjacent tissues (NAT) of early‐stage HCC patients. Furthermore, we aimed to integrate this data with previous transcriptomic data to identify potential subtypes of HCC and investigate potential therapeutic strategies.

## 2 MATERIALS AND METHODS

### 2.1 Clinical sample collection

HCC tissues were provided by the Department of Hepatobiliary Surgery of Xinqiao Hospital (Chongqing, China) from June 2016 to June 2021. Tumour tissues (TT) and normal adjacent tissues (NAT) were collected immediately after resection and stored at −80°C after freezing in liquid nitrogen. A total of 127 paired HCC samples were collected for analysis. Out of these, 80 samples were single solitary lesions restricted to a segment. The paired tissues were filtered based on the following criteria: (1) no anti‐cancer treatment was given before hepatectomy; (2) each subject had only one malignant lesion of HCC identified by an expert diagnostician using Computed Tomography; and (3) both the TT and NAT were from the same liver segment. Sample quality was ensured through the examination of each tumour and normal adjacent tissue by an expert pathologist. The pathologist confirmed that the samples met the following criteria: (1) they were histopathologically defined as HCC; (2) tumour tissues had a tumour purity of over 90%; (3) normal adjacent tissues did not contain any tumour cells. After applying these filters, we obtained 39 paired HCC samples for multiomics analysis. These included 3 paired samples from S1, 6 paired samples from S8 and 5 paired samples from S2‐S7. Additionally, two fresh HCC samples from S6 and S1 segments were collected for patient‐derived organoids (PDO) assay, and five paraffin‐embedded HCC tissues were obtained for IHC using the same criteria.

### 2.2 Protein extraction, digestion and liquid chromatography‐tandem mass spectrometer (LC‐MS/MS) analysis

Detailed materials and methods were available in Supporting Information. Briefly, the tissue samples underwent three washes in PBS to remove blood and debris. Then, the tissues were minced and lysed in 8 M urea buffer before being sonicated. The lysates were centrifuged and the resulting supernatant mixture was collected. After determining the mixture concentration, 100 µg supernatant were reduced with 10 mM dithiothreitol and alkylated with 10 mM iodoacetamide. Subsequently, the protein mixtures were digested with trypsin using the filter‐aided sample preparation (FASP) method. The digested samples were collected by centrifugation. Then, we used the Q Exactive HF‐X tandem MS/MS in conjunction with a LC system and a matched nano‐electrospray ion source for peptide analysis to map the proteome atlas. Meanwhile, HEK293T cell samples were run and Spearman's correlation coefficient was computed for assessing data quality.

### 2.3 Peptide identification and protein quantification

The proteomic platform (https://phenomics.fudan.edu.cn/firmiana/) was used for analysing MS raw files. Then the searched results were filtered to 1% protein‐level FDR with the target‐decoy strategy.^11^ Detailed parameter settings were available in Supporting Information.

### 2.4 Extraction of lipids and metabolites

After thawing the tissues, each of them was sampled 30 mg and transferred to a 1.5 mL centrifuge tube. A precooled methanol/water solution was added and vortexed for homogenise the tissue. After methyl tert‐butyl ether (MTBE) added, the tube was vortexed, cooled, ultrasonicated and centrifuged sequentially, resulting in the separation of the solution into upper and lower layers. Detailed materials and methods were available in Supporting Information.

### 2.5 LC‐MS/MS analysis of lipids

The mobile phase A and B are identical in both of negative and positive mode. Detailed materials and methods were available in Supporting Information. Briefly, the sample was separated using the microflow rate ultra‐high performance LC system. After chromatographic column equilibrated, the samples were delivered to the column and analysed using the Q Exactive HF‐X MS system. The lipids were detected in both positive and negative ion modes.

### 2.6 Lipid identification and quantification

Progenesis QI software was utilised for processing raw MS data, and lipid‐MAPS was utilised for database searching. Specifically, the missing values of searched results were imputed using the recommended methods. The search parameters and methods were descripted in detail in Supporting Information. Finally, the obtained high‐quality data were annotated using the LIPID MAPS database.

### 2.7 LC‐MS/MS analysis of metabolites

The mobile phase A and B are different in negative and positive mode, which were available in Supporting Information. Briefly, the samples were separated using the microflow rate using the Nexera UHPLC LC‐30A system. After chromatographic column equilibrated, the sample was delivered via an autosampler at a preset flow rate. Following separation on the chromatographic column, the samples were analysed using the Q Exactive HF‐X mass spectrometer in both positive and negative ion modes.

### 2.8 Metabolite identification and quantification

The MS raw files were analysed using Compound Discoverer for metabolite identification and quantification. The chemical standards and manually curated compound list was identified by our in‐house metabolite library and databases. For further annotation, the identified compounds were also searched against human metabolome database (HMDB), PubChem Compound database, Chemical Entities of Biological Interest database and Kyoto Encyclopedia of Genes and Genomes (KEGG) compound database.

### 2.9 Immune characterisation identification

The abundances of immune and stromal cell types for HCC paired samples were estimated using xCell (https://xcell.ucsf.edu/) at protein level.^12^ In this study, we utilised 39 paired HCC samples with proteomics data to identify immune characterisation. The same methods were also used in our previous study.^13^

### 2.10 Immunohistochemistry (IHC)

HCC samples were fixed in neutral formaldehyde and embedded in paraffin. Then, they were cut into 4 µm sections. Immunohistochemistry was performed on these sections using previously described methods.^14^ The slides were stained with primary antibodies: CD63 (OTI2G6, OriGene), S100A9 (OIT3F8, OriGene), RPRD1B (OTI1C7, OriGene), ALOX5 (OTI3F1, OriGene), PD‐L1 (66248‐1‐Ig, Proteintech), HSF1 (67189‐1‐Ig, Proteintech), RAN (67500‐1‐Ig, Proteintech), XPO1/CRM1 (D6V7N, CST), MPO (ZA‐0197, ZSGB) and CD8 (ZA‐0508, ZSGB). The slides were then imaged under a light microscope (BX63, Olympus Microsystems).

### 2.11 PDOs culture and viability assay

The HCC samples used in this study were obtained from two patients, as described in Supporting Information. To obtain single cells, the tumours were minced and digested. The organoids were then maintained in a 3D culture system and seeded in a 24‐well plate. These organoids were treated with KPT185 and Zileuton at the indicated concentrations for a duration of 6 days. Images were captured using an inverted microscope system. All the PDOs used in this study were provided by K2 Oncology Inc, Beijing.

### 2.12 Survival analysis

Overall survival (OS) of Clinical Proteomic Tumour Analysis Consortium (CPTAC) patients was analysed using Kaplan–Meier survival curves. *p* < .05 was considered as statistically significant. Before conducting the log‐rank test for each marker, survminer R package (version 0.2.4) with maxstat (maximally selected rank statistics) was utilised to determine the optimal cutpoint for the selected samples, following a previous study.^13^ Based on this optimal cutpoint, we calculated OS curves using Kaplan–Meier analysis combined with log‐rank test.

### 2.13 Statistical analysis

To compare continuous variables with different distributions, we performed Wilcoxon's test, Kruskal–Wallis test and Student's *t*‐test. All tests were two‐sided, and *p* < .05 was determined as a significance level, unless otherwise specified. Statistical analyses were conducted using R software (version 4.0.2) and GraphPad Prism8 software.

## 3 RESULTS

### 3.1 Multiomics profiling of segment‐resolved HCC

We enrolled 39 paired early‐stage HCC tissue samples (Table S1) and performed segment‐resolved untargeted proteomics, metabolomics and lipidomics profiling, which were then subjected to bioinformatic integration analysis (Figure 1A). The mass spectrometry (MS) platform demonstrated consistent stability in the quality control (QC) samples with an average correlation coefficient of 0.98 (range, 0.95–0.99) (Figure S1A). In comparison to the CPTAC HCC cohort, both cohorts detected a total of 8556 proteins. Specifically, 2659 proteins were identified in our HCC cohort, while the CPTAC cohort identified 1693 proteins (Figure 1B). We identified a total of 7852 common proteins between the TT and NAT samples. Additionally, we found 2795 proteins specifically detected in the TT sample and 567 proteins specifically detected in the NAT sample (Figure S1B). The number of detected proteins in TT samples (median, 7268) was significantly higher than that in the paired NAT samples (median, 5455), especially in the right lobe of the liver (*p* < 3.1e‐14; Figures 1C and S1C). This suggests a high degree of heterogeneity in HCC samples. The investigation conducted by the Human Protein Atlas (HPA) revealed that the TT samples exhibited a higher upregulation of subcellular proteins in comparison to the NAT samples (Figure 1D). Conversely, a significant downregulation of liver‐specific proteins, as annotated in the HPA, was observed in tumours (Figure S1D). This suggests a loss of liver identity as a characteristic in HCC.

To comprehensively characterise the multiomics profile of HCC, we conducted simultaneous analysis of the metabolome and lipidome in paired HCC samples. Consistency assessments were performed separately for metabolome and lipidome analysis, following QC workflow similar to that used in proteome analysis (Figure S1E and F). Our analysis revealed that the positive‐ion model of LC‐MS/MS identified a larger number of metabolites, including lipids, compared to the negative‐ion model (Figure 1E). We annotated a total of 3381 lipids and 1029 other metabolites by combining redundant metabolites with the same compound name (Figure 1F, Table S2). For the investigation of proteomics alterations in TT compared to NAT samples, we utilised 8536 proteins filtered by the Firmiana platform for further analysis (as described in the methods, Table S3). Our analysis revealed a noticeable difference in the proteomes between TT and NAT groups, as shown by the principal component analysis (PCA) (Figure S1G). The elliptical shadow, representing the 95% confidence interval (CI), was significantly larger in TT than in NAT, indicating high heterogeneity in HCC samples. Interestingly, similar results were observed in the orthogonal partial least‐squares discrimination analysis (OPLS‐DA) of metabolomes and lipidomes (Figure S1H and I).

### 3.2 Integration analysis of paired HCC samples

**FIGURE 2:**
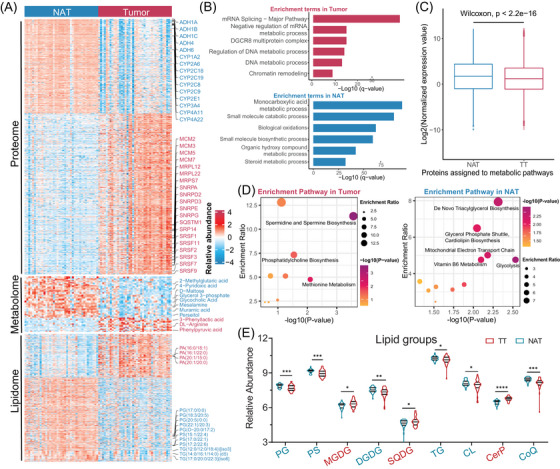
Multiomics insight analysis of the paired HCC samples. (A) Heatmap shows the profiles of proteins, metabolites and lipids in TT and NAT samples. (B) Bar plot represents the enrichment terms based on the dysregulated proteins between TT and NAT. (C) Box plot of the relative expression of the proteins assigned to metabolic pathway between NAT and TT groups. The metabolic associated proteins obtain from KEGG pathway dataset. *p* Value was calculated using the two‐sided Wilcoxon rank‐sum test. (D) Scatter plot presentation of the enrichment pathway based on the dysregulated metabolites between TT and NAT. (E) Violin plot of the dysregulated lipid groups in TT and NAT. *p* Value was calculated using the two‐sided *t*‐test based on the log2 transformed data. *p < .05, **p < .01, ***p < .001. CerP, ceramide 1‐phosphates; CL, cardiolipin; CoQ, coenzyme Q; DGDG, digalactosyl diacylglycerol; MGDG, monogalactosyl diacylglycerol; PG, phosphatidylglycerol; PS, phosphatidylserine; SQDG, sulphoquinovosyl diacylglycerol; TG, triglyceride.

A total of 908 different expression proteins (DEPs) were identified in TT compared to NAT (adjusted *p* value < .05, |log_2_FC| > 1). Among these, 571 were upregulated, while 337 were downregulated (Figure 2A, up‐panel, Table S4). It is worth noting that TT samples showed upregulation of proliferation‐related proteins such as mini‐chromosome maintenance proteins (MCMs), mitochondrial ribosomal proteins (MRPs), small nuclear ribonucleoprotein polypeptide (SNRPs) and serine and arginine rich splicing factor (SARSFs). In contrast, NAT samples exhibited upregulation of cytochrome P450 family proteins (CYPs) and alcohol dehydrogenases (ADHs), which are associated with material metabolism processes. Enrichment analysis consistently revealed that the DEPs upregulated in TT were primarily associated with proliferation‐related processes, such as mRNA splicing and DNA metabolic processes. On the other hand, the proteins upregulated in NAT were enriched in metabolic processes, including monocarboxylic acid metabolic process, small molecule catabolic process and biological oxidation (Figure 2B). Furthermore, the proteins assigned to metabolic pathways, as identified through Kyoto Encyclopedia analysis of Genes and Genomes (KEGG) database, were significantly upregulated in the TT samples compared to the NAT samples (*p* value < 2.2e‐16) (Figure 2C).

In the analysis of metabolomics and lipidomics, the fraction of total (FOT) was used to indicate the normalised abundance of a specific small molecule in all samples. The results showed that in TT samples, there were 31 metabolites that were up‐enriched and 100 that were down‐enriched, while 218 lipids were up‐enriched and 418 were down‐enriched compared to NAT samples (adjusted *p* value < .05, |log_2_FC| > 1, VIP (variable importance in projection) > 1) (Figure 2A, middle and down panel, Table S5). Based on the enrichment analysis, NAT samples exhibited a higher number of upregulated metabolic small molecules, suggesting increased metabolic processes such as glycolysis, triacylglycerol biosynthesis and mitochondrial electron transport chain compared to TT samples (Figure 2D). After merging lipids into the superclass, the NAT samples exhibited significantly higher levels of up‐enriched lipid groups, including phosphatidylglycerol (PG), phosphatidylserine (PS), triglyceride (TG), cardiolipin (CL), coenzyme Q (CoQ) and digalactosyl diacylglycerol (DGDG). On the other hand, monogalactosyl diacylglycerol (MGDG), sulphoquinovosyl diacylglycerol (SQDG) and ceramide 1‐phosphates (CerP) showed remarkable up‐enrichment in the TT samples (Figure 2E).

### 3.3 Segment‐resolved multiomics landscape of NAT

To analyse the multiomics landscape of the nonalcoholic steatohepatitis (NAT) from each liver segment, we initially employed the Kruskal–Wallis test (*p* < .05) to compute and filter 1209 DEPs from 8 segments. Utilising these DEPs, we conducted a correlation analysis and segregated the NAT samples from the 8 liver segments into two groups, which largely corresponded to the left and right lobes of the liver (Figure S2A). Subsequently, we performed an unsupervised hierarchical clustering of these DEPs, resulting in the classification of NAT samples into two clusters: cluster 1, mainly representing the left lobe (including S1–S4), and cluster 2, corresponding to the right lobe (consisting of S5‐S8) (Figure S2B).

**FIGURE 3:**
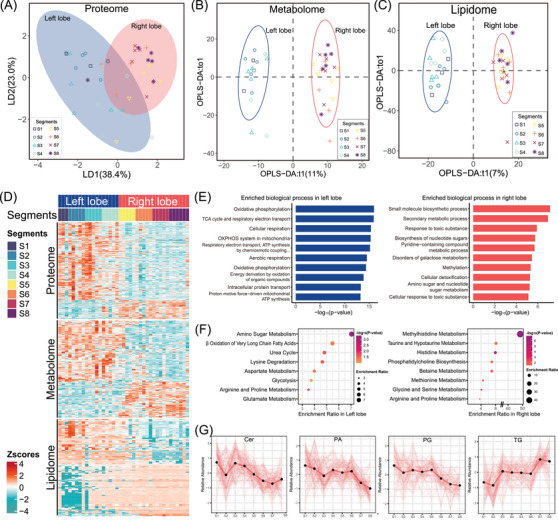
Multiomics landscape of NAT samples. (A) Linear discriminant analysis (LDA) in the proteomes of NAT samples largely classifies into left lobe including S1 (same in the following) and right lobe of liver. The elliptical shadow represented 95% confidence interval (95% CI). (B, C) Orthogonal partial least‐squares discrimination analysis (OPLS‐DA) in the metabolomes (B) and lipidomes (C) of NAT samples reveals a clear division between the left and right lobes. Left and right lobe samples are coloured in blue and orange, respectively. (D) Heatmap shows the dysregulated proteomes, metabolomes and lipidomes of NAT samples between the left and right lobes. (E) Enrichment analysis of DEPs in left lobe coloured blue and right lobe coloured orange, respectively. (F) MSEA of dysregulated metabolomes in left lobe and right lobe, respectively. (G) Line charts show the relative abundances of four lipid groups in the 8 segments of liver. Cer, ceramide; MSEA, metabolite set enrichment analysis; NAT, normal adjacent tissues; PA, phosphatidic acid; PG, phosphatidylglycerol; TG, triacylglycerol.

To compare the multiomics profiling of the left and right lobe of the liver, we employed linear discriminant analysis (LDA) to identify linear combinations of the NAT proteome data. The left lobe consisted of 18 samples (including S1), while the right lobe consisted of 21 samples. The resulting data was then projected onto the first two linear discriminants, which accounted for 61.4% of the variance within the two lobes. Through proteomics analysis (Figure 3A), the NAT samples were generally classified into left and right lobes, the finding that was further confirmed in the OPLS‐DA of metabolomes and lipidomes (Figure 3B and C). Differential analysis revealed a higher number of upregulated proteins and small molecules in the left lobe compared to the right lobe (Figure 3D). Specifically, we identified 614 dysregulated proteins in the proteomes, with 461 proteins upregulated in the left lobe and 153 proteins upregulated in the right lobe (*p* < .05, |log_10_FC| > 0.5). Furthermore, there were 204 upregulated metabolic molecules in the left lobe and 111 in the right lobe (*p* < .05, |log_10_FC| > 0.5), along with 377 upregulated lipids in the left lobe and 358 in the right lobe (*p* < .05, |log_10_FC| > 2). The results of the enrichment analysis revealed that dysregulated proteins in the left lobe were primarily associated with energy metabolism processes. These processes specifically included oxidative phosphorylation, TCA cycle, cellular respiration and ATP synthesis. On the other hand, in the right lobe, small molecule metabolism processes such as small molecule biosynthetic process, secondary metabolic process, response to toxic substance and biosynthesis of nucleotide sugars were found to be significantly enriched (Figure 3E).

The results were validated using metabolite set enrichment analysis (MSEA) in metabolomic data. The left lobe of the liver showed enrichment in the processes of β oxidation of very long chain fatty acids and glycolysis, while the right lobe showed enrichment in methylhistidine metabolism, taurine and hypotaurine metabolism and histidine metabolism (Figure 3F). The study also observed significant variations in the abundance of lipid groups among the 8 segments of the liver. Specifically, the left lobe exhibited upregulation of ceramides (Cer), phosphatidic acids (PA) and PG, while the right lobe showed upregulation of TG, particularly in the S7 and S8 segments (Figure 3G). Overall, the segment‐resolved multiomics NAT data allowed for differentiation between the left and right lobes of the liver and identification of their distinct enriched profiles.

### 3.4 Proteomic stratification of early‐stage HCC

Due to the variability among individuals, the relative abundance of multiomics data was initially calculated on TT/NAT. However, characterising HCC through metabolomics analyses posed challenges due to the complex and diverse chemical structure of metabolites, including lipids, which participate in multiple pathways.^8^, ^15^ Therefore, for the analysis of early‐stage HCC, integration analysis was predominantly used for proteomic stratification, while metabolome and lipidome were employed for subsequent functional validation. We employed nonnegative matrix factorisation (NMF) clustering to classify subtypes of HCC based on proteome data. Our analysis identified three subtypes using both the cophenetic and silhouette indicators with the ‘Brunet’ algorithm and ‘Lee’ algorithm (Figure S3A and B), respectively. Furthermore, our clustering approach was consistent with the consensus clustering of HCC in the proteome consensus matrix (Figure S3C).

**FIGURE 4:**
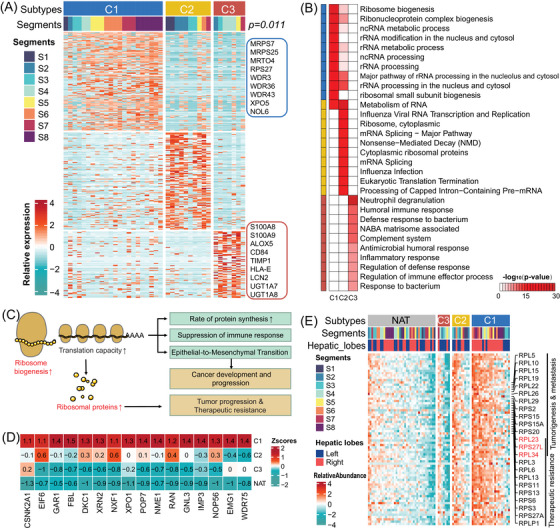
Protein abundance‐based clustering of HCC. (A) Relative expression of upregulated proteins (*p* < .05, log2FC > 4) in the three proteomic subtypes and associations of liver segments. Some typical proteins are labelled in rectangle. (B) The enriched top 10 GO terms (ranking in *p* value) of significantly differential expressed proteins among the proteomic subtypes. (C) Systematic diagram summarises the dysregulated ribosome biogenesis in cancer development and progression. (D) Dysregulated proteins related to ribosome biogenesis among proteomic subtypes and NAT samples. Colour of each cell shows *Z‐*score (log2 of relative abundance scaled by proteins’ SD) of the protein in that sample. (E) The relative expression of ribosomal proteins (RPs) among proteomic subtypes and NAT samples. Some RPs associated with tumour progression and therapeutic resistance are listed in the right of the diagram.

Based on proteomic stratification, the HCC samples were classified into three subtypes: C1, C2 and C3, which was consisted of 22, 10 and 7 cases, respectively (Figure 4A, Table S1). Gene Ontology (GO) annotation revealed that the C1 subtype is characterised by RNA metabolism‐related pathways, particularly the process of ribosome biogenesis (RB). This process involves proteins such as MRPS7, MRPS25, MRTO4, RPS27, WDR3, WDR36 and WDR43 (Figure 4B). Therefore, we defined the C1 subtype as the S‐RB subtype. On the other hand, the C3 subtype showed higher levels of proteins associated with immune response, specifically neutrophil degranulation (ND). These proteins include S100A8/9, CD84, TIMP1, HLA‐E and LCN2, and this subtype is referred to as the S‐ND subtype. Meanwhile, the C2 subtype displayed an intermediate phenotype, denoted as the S‐Im subtype, between the C1 and C3 subtypes.

### 3.5 Ribosome biogenesis‐driven HCC

Numerous recent studies have highlighted the important role of dysregulated ribosome biogenesis in epithelial–mesenchymal transition, cancer metastasis and therapeutic resistance.^16^, ^17^, ^18^ Hyperactivation of ribosome biogenesis can enhance translation capacity, resulting in increased protein synthesis for cell proliferation. Moreover, it can suppress the immune response by upregulating the expression of programmed death ligand 1 (PD‐L1) for cellular migration (Figure 4C). By leveraging the KEGG Pathway database, we identified 57 out of 82 proteins associated with ribosome biogenesis (RBs). Among these proteins, 16 exhibited significant relevance within the HCC proteome subtypes (Figure 4D). Furthermore, our analysis demonstrated that the heightened capacity for RNA translation leads to accelerated synthesis of ribosome proteins (RPs), which are known to be linked with tumour progression and therapeutic resistance.^16^ In this study, we measured and categorised 74 RPs into two main groups: ribosomal large subunits (RPLs) and ribosomal small subunits (RPSs). Examples of RPLs and RPSs include RPL15, RPL19, RPS6 and RPS15A, which have been associated with a negative prognosis in malignant neoplasms of the digestive system. Notably, the C1 subtype of hepatocellular carcinoma (HCC) showed the highest expression levels of RPs, followed by the C2 and C3 subtypes, while the lowest levels were observed in NAT (Figure 4E). In conclusion, the process of ribosome biogenesis plays a crucial role in the C1 subtype (S‐RB) of HCC.

**FIGURE 5:**
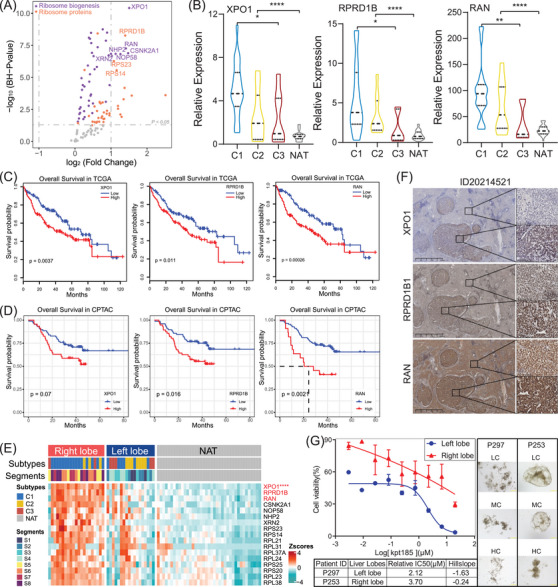
Elevated ribosome biogenesis promotes C1 subtype HCC progression. (A) Scatter plot depicts the ribosome biogenesis‐related proteins (RBs, violet points) and ribosome proteins (RPs, orange points) which were almost upregulated in TT compared with NAT group. The candidate proteins (adjust *p* value < 10^−5^, log_2_FC| > 1) are labelled in the graph. (B) Violin plot shows the candidate proteins of XPO1, RPRD1B1 and RAN significantly different expressed among the proteomic subtypes and NAT group. (C) Kaplan–Meier curves for overall survival based on the transcriptional expression of XPO1, RPRD1B1 and RAN in the TCGA HCC cohort, respectively. (D) Kaplan–Meier curves for overall survival based on the protein expression of XPO1, RPRD1B1, and RAN in the CPTAC HCC cohort, respectively. (E) Heatmap shows the relative expression of candidate RBs and RPs among left lobe, right lobe and NAT groups including the proteins of XPO1, RPRD1B1 and RAN. (F) IHC validates the relative expression of XPO1, RPRD1B1 and RAN in TT and NAT samples. (G) The PDOs obtained from the HCC tumour tissue are confirmed to be sensitive to XPO1 inhibitor (Kpt185) with IC_50_ of 2.12 and 3.7 in left and right lobe, respectively. The bright field images of PDO cells inhibited by Kpt185 were shown in the right. *p < .05, **p < .01, ***p < .001, ****p < .0001. HC, high concentration; LC, low concentration; MC, middle concentration; RAN, ras‐related nuclear protein; RPRD1B, regulation of nuclear pre‐mRNA domain‐containing protein 1b; and XPO1, exportin 1.

To identify potential therapy targets within ribosome biogenesis related processes, we examined the expression of candidate proteins from RBs and RPs in TT and NAT (Figure 5A). Among the HCC proteome subtypes and NAT, the C1 subtype exhibited the highest expression of three candidate proteins: exportin 1 (XPO1), regulation of nuclear pre‐mRNA domain‐containing protein 1b (RPRD1B), and ras‐related nuclear protein (RAN) (*p* < .0001, Figure 5B). In the TCGA HCC cohort, the Kaplan–Meier curves, along with the log‐rank test, demonstrated significant stratification of patients based on survival differences in XPO1, RPRD1B and RAN (Figure 5C). Furthermore, in an independent HCC cohort from the CPTAC portal, the survival differences were confirmed at the protein level for XPO1, RPRD1B and RAN (Figure 5D). Based on the proteome subtypes matched with hepatic lobes, we observed that the C1 subtype samples were predominantly located in the right lobe of the liver. Moreover, we identified three candidates with higher expression of RPs in the right lobe compared to the left lobe (*p* < .0001, Figure 5E). Consequently, we selected HCC samples from the right lobe for further immunohistochemistry (IHC) analysis. Our findings demonstrated a significant increase in the expression of XPO1, RPRD1B and RAN in TT compared to NAT (Figure 5F).

Previous studies have indicated that heat shock transcription factor 1 (HSF1) serves as a marker for a widespread metastatic program found in various cancers. This marker is upregulated due to enhanced ribosome activity and biogenesis.^17^, ^19^, ^20^ Consistent with the three candidate proteins mentioned earlier, it was observed that the expression of HSF1 protein was higher in TT compared to NAT (Figure S4A). The signalling mechanism of immune checkpoint PD‐L1/PD‐1 plays a crucial role in allowing cancer cells to evade detection and attack by T cells, thus enabling them to escape the immune system.^21^ Our study revealed that the expression of PD‐L1 was lower in TT compared to NAT (Figure S4A). Furthermore, we observed a high expression of CD8 (a marker of CD8^+^ T cells) and CD68 (a marker of macrophages) in NAT, while their presence was almost undetectable in the tumour. These findings were further validated in other independent HCC patients using the IHC method in paraffin sections (Figure S4B and C).

PDO assays were utilised in our study to identify potential targets for precise treatments.^22^ Our findings showed that the protein expression of XPO1, RAN or RPRD1B was higher in the right lobe compared to the left lobe (Figure 5E). Consequently, we selected HCC samples from the right lobe for further immunohistochemistry (IHC) analysis. Our findings demonstrated a significant increase in the expression of XPO1, RPRD1B, and RAN in TT compared to NAT (Figure 5F). Currently, selective inhibitors of XPO1 have been approved as a promising therapy for malignant tumours, but not RAN or RPRD1B.^23^ Therefore, we selected two HCC samples labelled KOLV‐057 and KO‐67220, which had primary lesions in the left lobe and right lobe respectively, for the PDO assays. As expected, the inhibitor Kpt185, which targets XPO1, significantly reduced the proliferation and survival of HCC cells, with an IC_50_ of 2.12 µM and 3.7 µM (Figure 5G), suggesting XPO1 as a target for C1 subtype of HCC.

### 3.6 Neutrophil degranulation‐driven HCC

**FIGURE 6:**
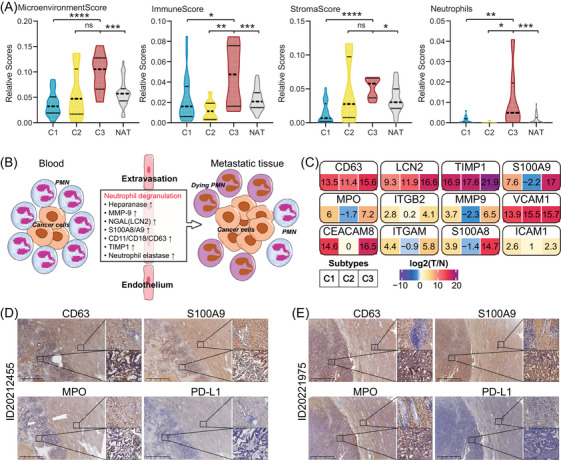
Neutrophil degranulation promotes HCC metastasis. (A) Violin plot shows the significantly different estimate scores of immune (including neutrophil granulocyte) and stroma across HCC subtypes and NAT samples. (B) Schematic diagram shows a plausible mechanism of neutrophil degranulation in cancer metastasis. (C) The proteins of neutrophil granulocyte differently expressed among the HCC proteome subtypes, such as CD63, LCN2, S100A9, MPO, ITGB2, MMP9, VCAM1, CEACAM8, ITGAM, S100A8 and ICAM1. (D, E) IHC analysis validates the relative expression of CD63, S100A9, MPO and PDL1 in TT and NAT samples from the patients of ID20212455 (D) and ID20221975 (E).

Recent studies have shown that neutrophil degranulation may promote cancer metastasis and potentially have an immunosuppressive role.^24^ In our research, we found a close association between the S‐ND (C3) subtype of hepatocellular carcinoma (HCC) and immune response, specifically neutrophil degranulation (Figure 4A and B). Consistent with our expectations, the xCell algorithm indicated higher scores for tumour microenvironment, immune response, stroma and neutrophils in the C3 subtype compared to the other subtypes and NAT (Figure 6A).

During the process of cancer metastasis, the exocytosis of neutrophil granules leads to changes in the neutrophil cell‐surface markers and the release of granule matrix proteins. These proteins include myeloperoxidase (MPO), CD63, CD18 (ITGB2), CD11 (ITGAM), TIMP1, heparinase (HPSE), MMP9, S100A8/9 and LCN2 (Figure 6B). Among these, the C3 subtype exhibits the highest levels of proteins associated with granule exocytosis, particularly CD63, S100A9 and MPO (Figure 6C). Furthermore, elevated expression levels of granule proteins derived from neutrophils and released cytokines are correlated with a poorer prognosis for patients with HCC (Figure S5). To validate these findings, we performed IHC analysis on paraffin sections from independent HCC patients. Consistently, we observed higher expression levels of CD63, S100A9, and MPO in TT samples compared to NAT samples (Figure 6D and E).

**FIGURE 7:**
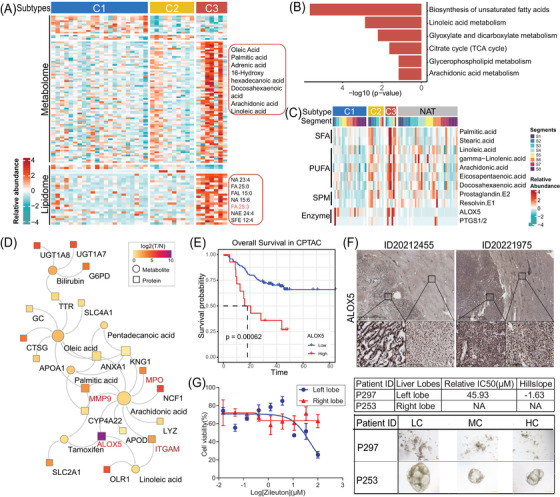
Dysregulated PUFA metabolism‐associated neutrophil degranulation facilitates HCC development. (A) Heatmap shows the dysregulation of metabolome and lipidome in the HCC subtypes. (B) MSEA of these upregulated metabolites in C3 subtype compared with other subtypes. (C) Heatmap shows the relative expression of SFA, PUFA, SPM and enzyme among the HCC subtypes and NAT. (D) The interaction of fatty acids with regulated proteins. The size of circles (metabolites) and squares (proteins) indicates the frequency of interaction. (E) Kaplan–Meier curves of overall survival for ALOX5 in CPTAC HCC cohort. (F) IHC analysis from two independent patients (ID20212455 and 20221975) validates the expression of ALOX5 in TT and NAT samples. (G) PDOs obtained from HCC tumour tissues are identified be sensitive to ALOX5i (Zileuton) with IC_50_ of 45.93 µM in left lobe but not in right lobe. HC, high concentration; LC, low concentration; MC, middle concentration; PDO, patient‐derived organoid; PMN, polymorphonuclear neutrophils.

The integrational analysis of multiomics was conducted to characterise the C3 subtype. The results indicated that the C3 subtype had a higher abundance of metabolites and lipids, specifically oleic acid, palmitic acid (PA), arachidonic acid (ARA) and linoleic acid (LA), in comparison to the C1 and C2 types (Figure 7A). To further investigate the function of the C3 subtype, an MSEA was performed. This analysis revealed a significant enrichment of the biosynthesis of unsaturated fatty acid, as well as LA and ARA metabolism, in the C3 subtype (Figure 7B).

The development and progression of liver cancer have been associated with fatty acid metabolism.^25^ In the de novo synthesis process, palmitic acid (PA) and stearic acid (SA) are produced by adding two carbons at a time to acetyl‐CoA. Polyunsaturated fatty acids (PUFAs) can be classified into two groups: ω−6 FAs (including LA, GLA and ARA) and ω−3 FAs (such as ALA, DHA and EPA) (Figure S6A). The C3 (S‐ND) subtype showed upregulation of several FAs, specialised pro‐resolving lipid mediators (SPMs), and enzymes involved in FA metabolism, including LA, ARA, prostaglandin E1/2 and ALOX5 (Figures 7C and S6B).

The FA metabolism is essential for the immune response of neutrophils.^26^ Our network analysis identified a close regulation of ARA metabolism by the enzyme ALOX5. This metabolism interacts with proteins involved in neutrophil degranulation, such as MMP9, MPO and ITGAM (Figure 7D). Correlation analysis revealed that the expression level of ALOX5 positively correlated with the expression of MMP9 (*R* = 0.67, *p* < .001), CD63 (*R* = 0.73, *p* < .001) and ITGAM (*R* = 0.86, *p* < .001) (Figure S7). Moreover, higher expression of ALOX5 was associated with a poorer prognosis for HCC patients in the CPTAC cohort (Figure 7E). The expression of ALOX5 was also validated in independent patients with tumours originating from the C3 subtype using the IHC method (Figure 7F).

Previous findings showed that ALOX5 expression was higher in the C3 subtype compared to other subtypes or segments (Figure 4A). Therefore, similar to the experiment conducted on the C1 (S‐RB) subtype HCC (as depicted in Figure 5G), we also used the immortalised cells of KOLV‐057 and KO‐67220 for the PDO drug sensitivity assays (Figure 7G). Despite using a higher drug concentration than Kpt185, Zileuton, a drug that inhibits the activity of ALOX5, was unable to reduce the proliferation of malignant cells obtained from the right lobe HCC tissue. Interestingly, Zileuton exhibited a significant decrease in the proliferation and survival of malignant cells immortalised from the left lobe HCC, with an IC_50_ value of 45.93 µM. These data demonstrate that ALOX5 is a potential target for C3 subtype of HCC.

## 4 DISCUSSION

The liver is a vital organ that plays a crucial role in maintaining homeostasis and regulating metabolism and immunity. With the advancement of more sensitive LC‐MS/MS techniques, there is now an opportunity for a more in‐depth examination of identified proteins, metabolites and lipids.^27^ In this study, we conducted a systematic analysis of the proteome profiling and metabolic characteristics of liver segments. We were able to accurately identify and quantify a larger number of proteins (No. = 11 215), metabolites (No. = 1029) and lipids (No. = 3381) compared to previous global untargeted tissue proteomics, metabolomics and lipidomics studies.^5^, ^6^, ^27^, ^28^, ^29^

Previously, hepatic segments that were histologically identical were traditionally considered to have the same function within the liver. However, it is now understood that these segments may differ in their microenvironment due to variations in the branches of the Glisson system. In this study, we aimed to investigate potential functional differences among different liver segments through segment‐resolved multiomics analysis. Our findings indicated that segments in the left lobe of the liver displayed characteristics associated with energy metabolism, including oxidative phosphorylation, the TCA cycle, respiratory electron transport, fatty acid oxidation and glycolysis. Conversely, segments in the right lobe showed a focus on small molecule metabolism, particularly amino acid metabolism. By comparing the HCC samples with NAT samples, we categorised the HCCs into three distinct subtypes: S‐RB, S‐Im and S‐ND. Notably, the HCCs in the right lobe, which can be broadly classified as S‐RB subtype, showed an upregulation of genes involved in the ribosome biogenesis process. In particular, XPO1, RAN and RPRD1B1 emerged as promising targets for ribosome biogenesis in right‐lobe HCCs. While selective inhibitors of XPO1 have already been approved for adjuvant therapy in other cancers, inhibitors for RAN and RPRD1B1 have not yet been developed.^23^ Our present study discovered that kpt185, an XPO1 inhibitor, significantly suppressed cell viability in the HCC PDO assays. These findings suggest that XPO1 inhibitors hold promise as a potential treatment option for HCC patients. However, further studies are required to confirm the relationship between the protein expression level of XPO1 and the effectiveness of XPO1 inhibitors in HCC treatments.

Recent research has extensively investigated the plasticity of neutrophils and their degranulation in the tumour microenvironment.^24^, ^26^, ^30^, ^31^ Our findings reveal a close association between the immune response process, specifically neutrophil degranulation, and the C3 (S‐ND) subtype of HCC. Furthermore, we observed that dysregulated PUFA metabolism can enhance the process of neutrophil degranulation. It is well‐established that the accumulation of neutrophils is stimulated by leukotriene B_4_ (LTB_4_) derived from ARA, which is catalysed by ALOX5.^30^ We found that the expression level of ALOX5 was higher in the S‐ND subtype of HCC compared to other subtypes, and this was associated with a poor prognosis in HCC patients. This result aligns with a previous proteomic study that also reports a high expression of ALOX5 in the immune subtype (S‐III) and its correlation with poor prognosis.^6^ In our current study, ALOX5i (Zileuton) effectively inhibits the perforation of malignant cells derived from the left lobe of HCC tissues, but not from the right lobe. This finding suggests that ALOX5 could be a potential target for S‐ND patients. However, it should be noted that only malignant cells, and not neutrophils, can be immortalised from HCC tissues in the PDO assay. Therefore, the therapeutic response of ALOX5i might be mediated through the protein expression level of ALOX5 in HCC cells rather than the accumulation of neutrophils. Further studies are required to establish the relationship between the efficacy of ALOX5i, neutrophil degranulation, and HCC prognosis.

In summary, our study delved into the intricate multiomic landscape of HCC paired samples. Notably, we observed a distinct classification of the liver into left lobe, characterised by energy metabolism, and right lobe, associated with small molecule metabolism. Through NAT reference profiling, we identified three subtypes of HCC: S‐RB, S‐Im and S‐ND. Our findings suggest that XPO1 could serve as a promising treatment target for S‐RB patients, while ALOX5i, linked to neutrophil degranulation, presents a novel avenue for treating S‐ND patients. However, it is important to acknowledge the limitations of our study, including the need for validation of HCC subtype categories in other cohorts, clarification of the detailed mechanisms underlying XPO1 and ALOX5 in HCC invasion and metastasis, and the necessity of clinical trials to confirm the efficacy of XPO1i and ALOX5i in treating HCC.

## 5 CONCLUSION

Through the integration of proteome, metabolome and lipidome data, we successfully established a reference profiling for HCC subtypes. This novel approach has the potential to offer more accurate and personalised treatment options for patients. Our findings may contribute to the advancement of multiomics‐driven precision medicine in the future.

## AUTHOR CONTRIBUTIONS

*Yongsheng Li and Shuai Yang*: Conceived and designed the project. *Shuai Yang, Jiangang Zhang and Lu Zheng*: Collected the human samples and clinical information. *Junying Chen*: Performed pathological examination. *Hui Gan*: Exanimated the liver segments. *Kai Li*: Performed the multiomics experiments. *Shuai Yang and Lingling Li*: Performed bioinformatic analyses. *Mei Yan and Chuanyin*: He performed IHC experiments. *Shuai Yang, Lu Zheng, Lingling Li, Jiangang Zhang, Jingchun Wang, Huakan Zhao, Yu Chen, Kai Li, Chen Ding and Yongsheng Li*: Discussed and interpreted the data. *Shuai Yang, Lingling Li and Yongsheng Li*: Wrote the manuscript. *Yongsheng Li*: Supervised the project.

## CONFLICT OF INTEREST STATEMENT

The authors declare that they have no competing interests.

## ETHICS STATEMENT

The human subjects were conducted in accordance with the principles outlined in the Declaration of Helsinki and approved by the Institutional Review Board of the Army Medical University and Chongqing University, China (Approval nos. AMUWEC2019304, CZLS2022114‐A and CZLS2023239‐A). This article does not contain any studies with animal subjects.

## Supporting information

Supporting Information

Supporting Information

Supporting Information

Supporting Information

Supporting Information

Supporting Information

Supporting Information

Supporting Information

Supporting Information

Supporting Information

Supporting Information

## Data Availability

The data supporting the findings of this study, including clinical information, proteome, lipidome and metabolome, can be found in the paper and its extended data. Alternatively, the mass spectrometry proteomics data have been deposited to the ProteomeXchange Consortium (https://proteomecentral.proteomexchange.org) via the iProX partner repository with the dataset identifier PXD050969.^32^, ^33^ Raw data of metabolome and lipidome have been deposited in the OMIX, China National Center for Bioinformation/Beijing Institute of Genomics, Chinese Academy of Sciences (https://ngdc.cncb.ac.cn/omix: accession no. OMIX006116 and OMIX006124).^34^, ^35^ The TCGA RNA‐seq data is publicly available at the Genomic Data Commons Data Portal (CDC, http://www.ncbi.nlm.nih.gov/geo). The data of CPTAC HCC cohort can be viewed in NODE (https://www.biosino.org/node) by searching for the accession (OEP000321) in the text search box or through the following URL: https://www.biosino.org/node/project/detail/OEP000321. R code for these analyses is available online via https://github.com/Andy‐0801/hcc_proteome.git.
