# Supplementary material for: Integrative multiomics analysis identifies molecular subtypes and potential targets of hepatocellular carcinoma
Source: Clin Transl Med. 2024 May 28;14(6):e1727. doi: 10.1002/ctm2.1727 (PMC11131356; doi:10.1002/ctm2.1727)
Supplement: Supplementary file 1 — Supporting Information [file CTM2-14-e1727-s002.docx]

### Supplementary figures


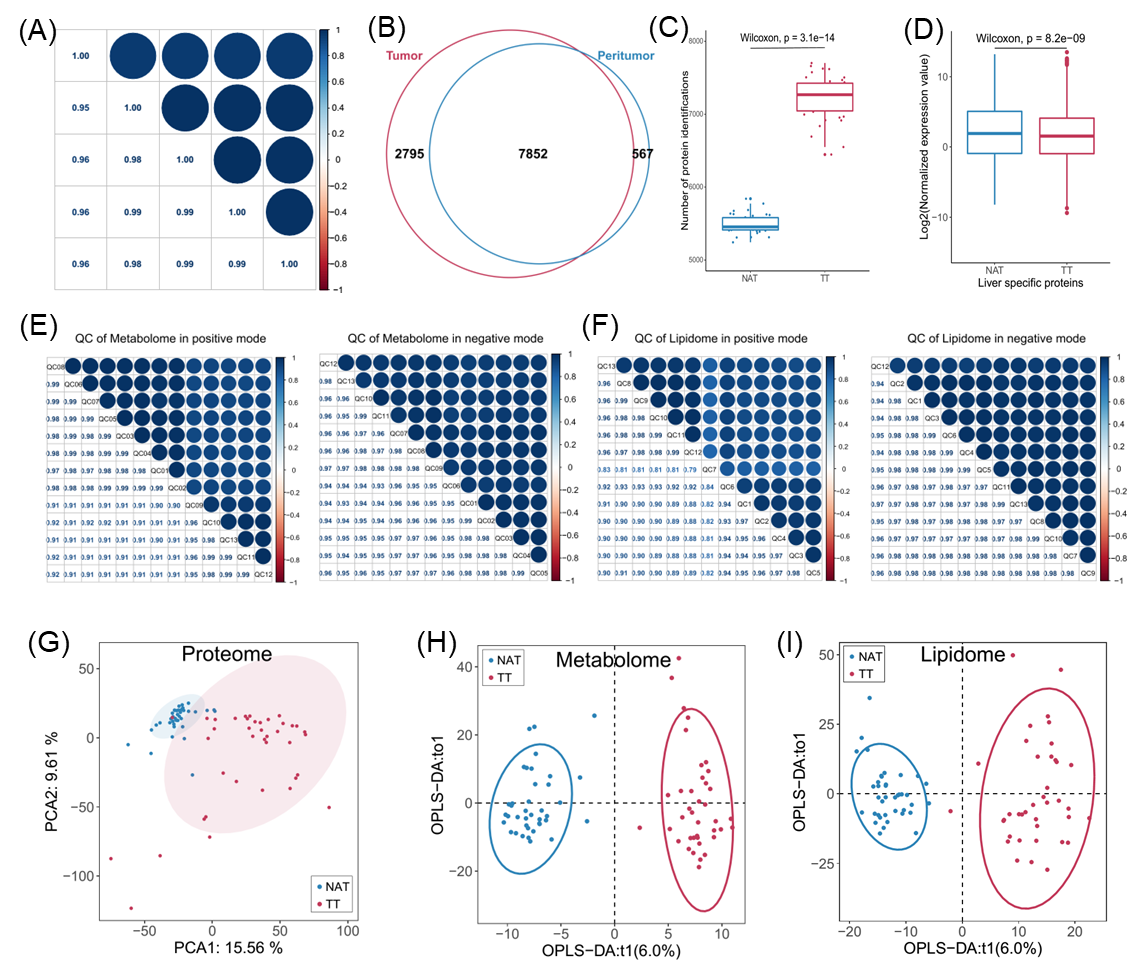


**Figure S1**. General analysis of the paired HCC and quality control samples. (A) Longitudinal quality control of MS using tryptic digests of HEK293T cells. The bottom-left half of the panel represents the pairwise Pearson’s correlation coefficients of the samples (two-sided Pearson’s correlation test), and the top-right half of the panel depicts the pairwise scatter plots from the same comparison. (B) Venn diagram depicts the overlap proteins identified in the TT and NAT samples. (C) Boxplot shows the number of identified proteins of the TT and NAT samples, respectively. *P* value was calculated using the two-sided Wilcoxon rank-sum test. (D) Box plot of log2-transformed relative expression of the liver-specific protein between NAT and TT groups. *P* value was calculated using the two-sided Wilcoxon rank-sum test. (E–F) QC samples for metabolome (E) and lipidome (F) were respectively analyzed in positive and negative model using the same method for the quality control of proteome analysis. The bottom-left half of the panel represents the pairwise Pearson’s correlation coefficients of the samples (two-sided Pearson’s correlation test), and the top-right half of the panel depicts the pairwise scatter plots from the same comparison. G. Principle component analysis (PCA) of the proteomic data largely separated tumor samples from NAT samples and the elliptical shadow represented 95% confidence interval (95% CI). H and I Orthogonal partial least-squares discrimination analysis (OPLS-DA) of the metabolomes (H) and lipidomes (I) in the TT and NAT samples. TT: tumor tissues; NAT: normal adjacent tissues. Tumor and normal samples are colored in red and blue, respectively.


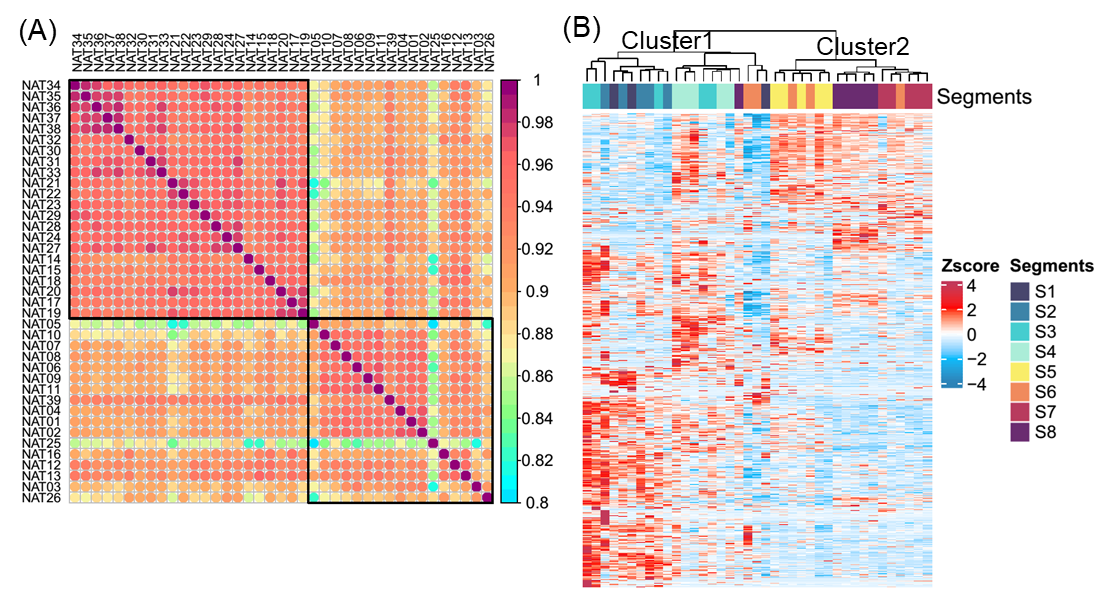


**Figure S2**. Two clusters of the NAT samples. (A) Correlation analysis of the NAT samples classified into two groups. (B) Heatmap shows the unsupervised hierarchical clustering of NAT samples were divided into two clusters.


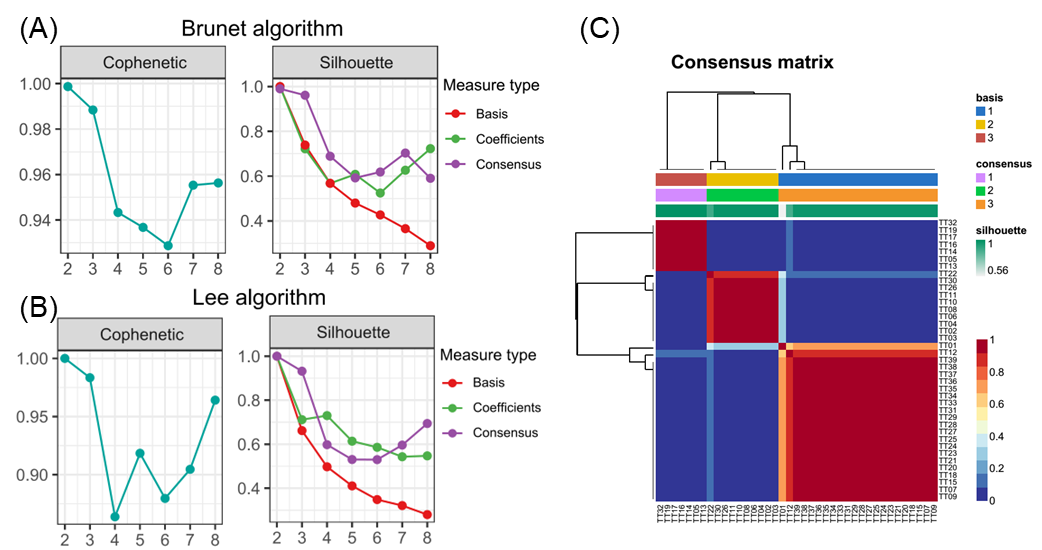


**Figure S3**. HCC classified into 3 subtypes based on segment-resolved proteomics analysis. (A–B) The standard NMF method of “brunet” (A) and “Lee” (B) was selected to identify HCC proteome subtypes, respectively. The cophenetic and silhouette indicators were plotted for the optimal rank of 3. (C) Heatmap shows the proteome consensus matrix with the clustering number of 3 in basis (accessing NMF factors) and consensus clustering methods.


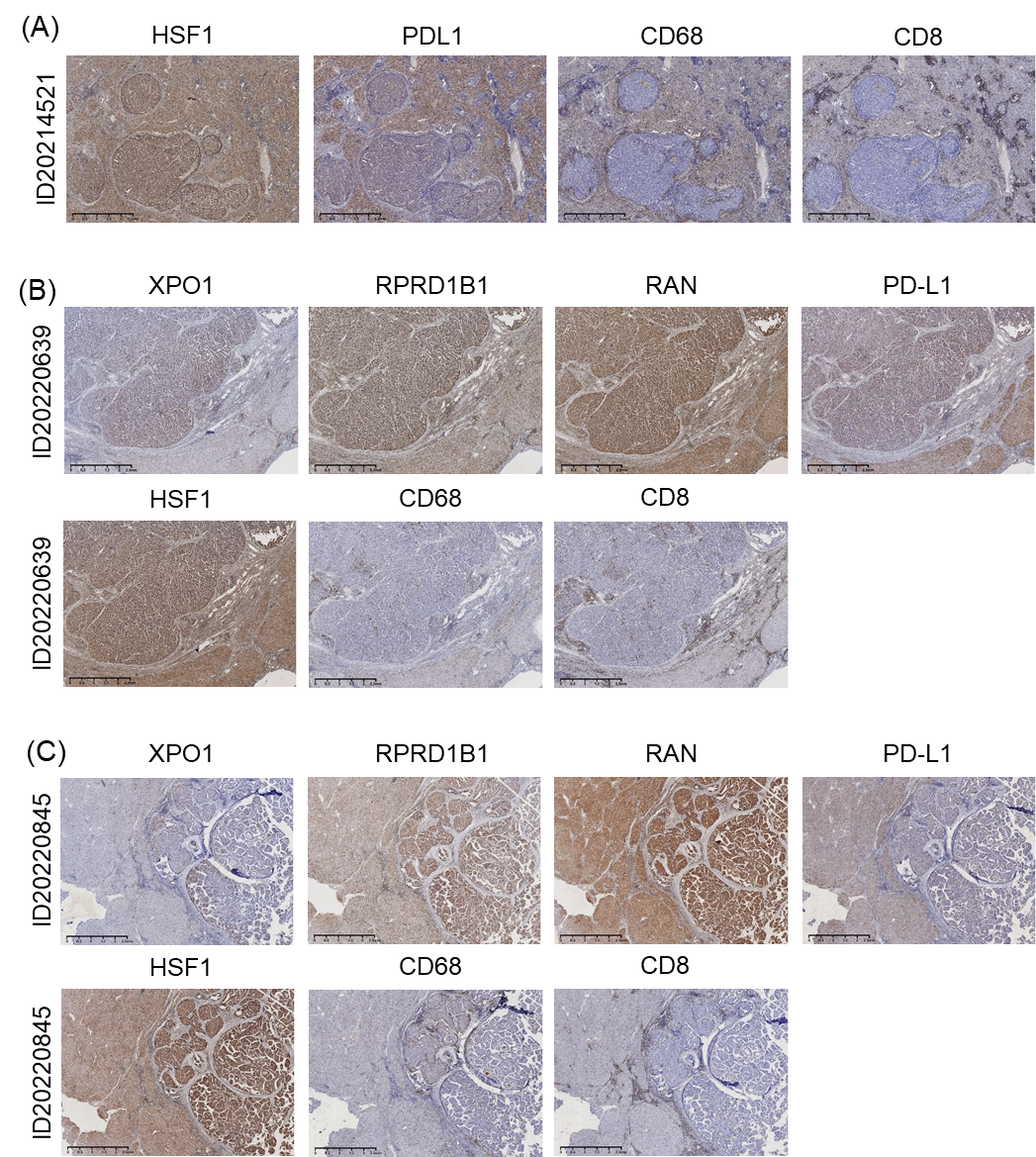


**Figure S4**. Representative protein expression involving ribosome biogenesis and immune markers in TT and NAT samples. (A) IHC estimating the expression of HSF1 and immune markers, such as PD-L1, CD8, and CD68 in TT and NAT samples. B and (C) IHC of XPO1, RPRD1B1, RAN, PD-L1, HSF1, CD8 and CD68 in TT and NAT samples from the patients with ID20220639 (B) and ID20220845 (C).


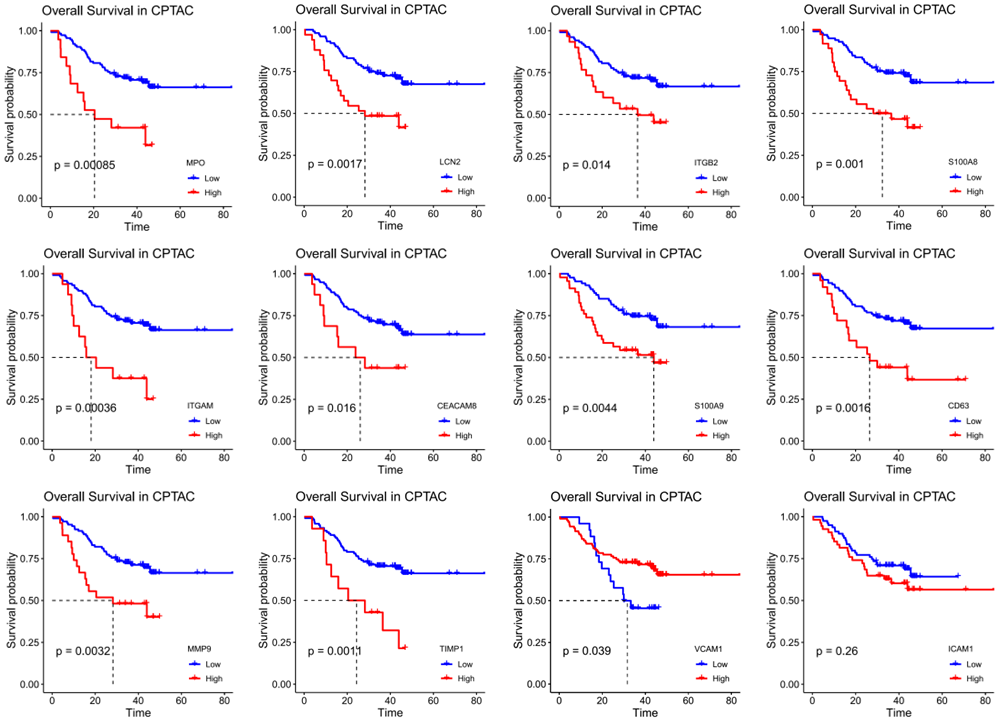


**Figure S5**. Neutrophil degranulation is an important regulator for HCC. Kaplan-Meier curves of overall survival for the proteins of neutrophil degranulation in CPTAC HCC cohort, respectively.


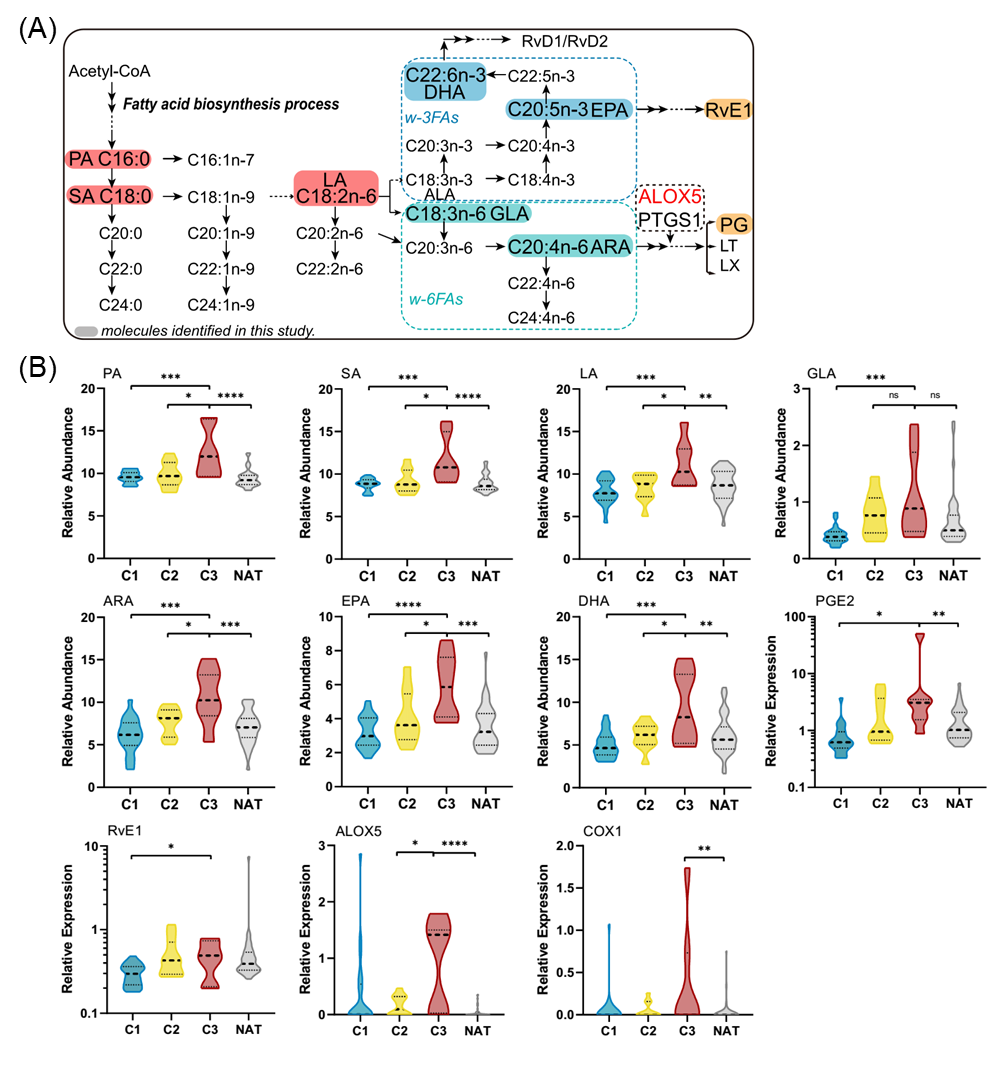


**Figure S6**. The relative abundance of intermediate metabolites in the process of FA metabolism. (A) Schematic diagram shows the mainly intermediate metabolites in the biosynthesis of fatty acid and some terminal products of catabolism. The arrow represents the direction of metabolism, the dotted arrow means the process cannot occur in human body. (B) Violinplot shows the significantly different abundance of metabolites or lipids across HCC subtypes and NAT samples. *P<0.05, **P<0.01, ***P<0.001, ****P<0.0001. PA: palmitic acid, SA: stearic acid, LA: linoleic acid, GLA: γ- linoleic acid, ARA: arachidonic acid, DHA: docosahexaenoic acid, EPA: eicosapentaenoic acid, RvD1/2: resolvin D1/2, RvE1/2: resolvin E1/2, PG: prostaglandin, LT: leukotriene, LX: thromboxan(E) SFA: saturated fatty acid, PUFA: polyunsaturated fatty acid, SPM: specialized pro-resolving lipid mediators.


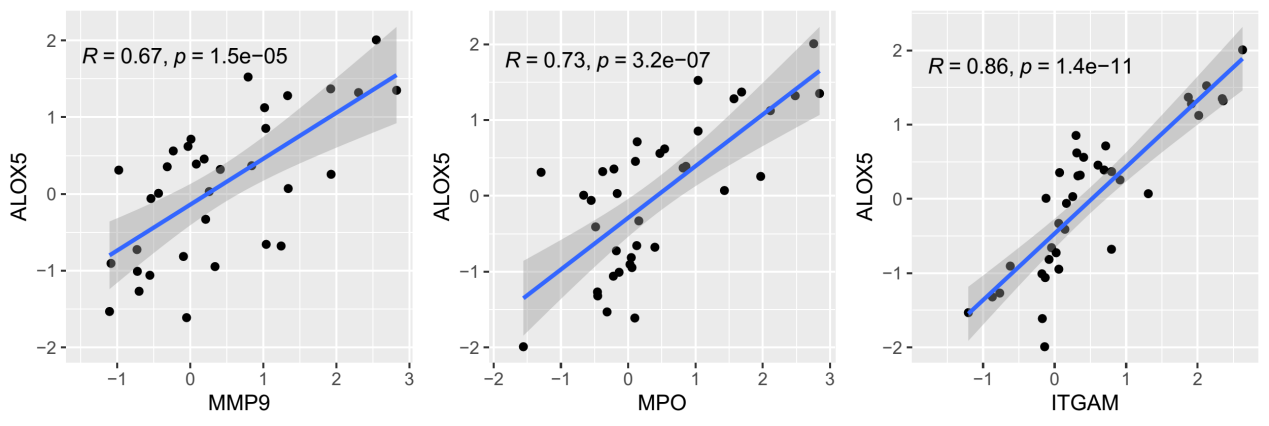


**Figure S7.** FA metabolism may regulate neutrophil degranulation. Correlation analysis between the relative expression of ALOX5 with MMP9, MPO and ITGAM, respectively.

### Supplemental tables

**Supplemental Table 1. Clinicopathologic information of 39 enrolled patients.** The measuring units and reference ranges were list in the brackets.

| **Tumor tissue (TT) ID** | **Normal adjacent tissue (NAT) ID** | **Gender** | **Age (year)** | **BMI (kg/m2)** | **Segments** | **Grade** | **TBIL (5.13～22.24μM/L)** | **AFP(0-10ng/mL)** | **HBV-DNA (IU/mL)** | **HBsAg1 (IU/mL)** | **HCC subtypes** |
| --- | --- | --- | --- | --- | --- | --- | --- | --- | --- | --- | --- |
| TT01 | NAT01 | M | 54 | 29.41 | S1 | G2 | 15.8 | 1.86 | <20 | >250 | C1 |
| TT02 | NAT02 | F | 31 | 23.19 | S1 | - | 23.8 | 2000 | 1740 | 58.82 | C2 |
| TT03 | NAT03 | M | 51 | 25.00 | S1 | - | 11.4 | 9.73 | <20 | 233.62 | C2 |
| TT04 | NAT04 | F | 44 | 27.38 | S2 | G3 | 12.6 | 10.15 | 77 | >250 | C2 |
| TT05 | NAT05 | M | 57 | 24.52 | S2 | G1 | 17.4 | 8 | <20 | 0 | C3 |
| TT06 | NAT06 | M | 57 | 26.31 | S2 | G2 | 20.1 | 2.49 | <20 | ＞250 | C2 |
| TT07 | NAT07 | M | 59 | 21.27 | S2 | G2 | 18.7 | 2000 | 12100 | ＞250 | C1 |
| TT08 | NAT08 | M | 49 | 20.14 | S2 | G2-G3 | 35.1 | 1850.21 | 578000 | ＞250 | C2 |
| TT09 | NAT09 | F | 62 | 26.44 | S3 | G1-G2 | 17.7 | 7.63 | 2070000 | ＞250 | C1 |
| TT10 | NAT10 | F | 51 | 20.22 | S3 | G2 | 25.1 | 209.53 | 368000 | >250 | C2 |
| TT11 | NAT11 | M | 50 | 23.45 | S3 | G1 | 27.5 | 4.3 | 138 | ＞250 | C2 |
| TT12 | NAT12 | F | 56 | 31.28 | S3 | G1-G2 | 16.3 | 2000 |  | 0 | C1 |
| TT13 | NAT13 | M | 48 | 24.38 | S3 | G3 | 20 | 3.69 | 123 | ＞250 | C3 |
| TT14 | NAT14 | M | 65 | 26.89 | S4 | G2 | 11.6 | 107.48 | ＜20 | 206.75 | C3 |
| TT15 | NAT15 | M | 55 | 23.99 | S4 | G2-G3 | 23.6 | 6.21 | 49100 | ＞250 | C1 |
| TT16 | NAT16 | M | 56 | 21.96 | S4 | G2-G3 | 18.1 | 2000 | 202 | ＞250 | C3 |
| TT17 | NAT17 | M | 49 | 18.04 | S4 | G3 | 17.6 | 1.67 | 72.7 | 193.34 | C3 |
| TT18 | NAT18 | M | 64 | 23.63 | S4 | G2 | 26.7 | 10.43 | 91900 | ＞250 | C1 |
| TT19 | NAT19 | F | 56 | 22.99 | S5 | G2-G3 | 11.8 | 44.14 | 822 | ＞250 | C3 |
| TT20 | NAT20 | M | 53 | 22.48 | S5 | G2 | 17.9 | 10.41 | 97300 | ＞250 | C1 |
| TT21 | NAT21 | F | 71 | 24.87 | S5 | G1-G2 | 15.3 | 11.94 | <20 | 0.03 | C1 |
| TT22 | NAT22 | M | 51 | 22.19 | S5 | G2 | 1.54 | 94.82 | 442000 | >250 | C2 |
| TT23 | NAT23 | M | 63 | 22.19 | S5 | G2-G3 | 8.7 | 798.1 | 132 | >250 | C1 |
| TT24 | NAT24 | M | 42 | 22.48 | S6 | G2-G3 | 18.7 | 126.53 | 611000 | >250 | C1 |
| TT25 | NAT25 | M | 76 | 20.34 | S6 | G2-G3 | 8.5 | 2000 |  | 10.18 | C1 |
| TT26 | NAT26 | F | 38 | 18.49 | S6 | G2-G3 | 10.9 | 1175.33 | 3050 | >250 | C2 |
| TT27 | NAT27 | M | 53 | 21.53 | S6 | G2 | 21.2 | 507.98 | <20 | >250 | C1 |
| TT28 | NAT28 | F | 25 | 18.55 | S6 | G2 | 12.5 | 2000 | 4270000 | 197.36 | C1 |
| TT29 | NAT29 | M | 47 | 20.72 | S7 | G2 | 11.4 | 4.14 |  | ＞250 | C1 |
| TT30 | NAT30 | M | 63 | 27.72 | S7 | G1-G2 | 20.3 | 2.03 | <20 | 0 | C2 |
| TT31 | NAT31 | M | 53 | 25.40 | S7 | G2 | 11 | 8.08 | <20 | 2.1 | C1 |
| TT32 | NAT32 | M | 67 | 21.48 | S7 | G2-G3 | 14.2 | 48.46 | 6140 | ＞250 | C3 |
| TT33 | NAT33 | M | 52 | 21.78 | S7 | G2 | 17.6 | 2000 | 770000 | ＞250 | C1 |
| TT34 | NAT34 | M | 42 | 23.14 | S8 | G2-G3 | 16.3 | 13.97 | 7.15 | 152.19 | C1 |
| TT35 | NAT35 | M | 48 | 23.32 | S8 | G1-G2 | 19.8 |  | 2440 | ＞250 | C1 |
| TT36 | NAT36 | M | 49 | 25.81 | S8 | G2-G3 | 9.4 | 7.75 | <20 | 213.66 | C1 |
| TT37 | NAT37 | M | 80 | 24.22 | S8 | G2-G3 | 19.6 | 2000 | <20 | 0 | C1 |
| TT38 | NAT38 | M | 53 | 23.62 | S8 | G2-G3 | 24.8 | 2000 | 282000 | ＞250 | C1 |
| TT39 | NAT39 | M | 24 | 24.09 | S8 | G2-G3 | 10.8 | 548.48 | 1820000 | ＞250 | C1 |
