# Supplementary material for: Integrative multiomics analysis identifies molecular subtypes and potential targets of hepatocellular carcinoma
Source: Clin Transl Med. 2024 May 28;14(6):e1727. doi: 10.1002/ctm2.1727 (PMC11131356; doi:10.1002/ctm2.1727)
Supplement: Supplementary file 2 — Supporting Information [file CTM2-14-e1727-s007.docx]

#### Funding Information

This work was supported by the Major International (Regional) Joint Research Program of the National Natural Science Foundation of China (No. 81920108027), Funding for National Clinical Key Specialty (Oncology), Funding for Chongqing Young Expert Studio, and Funding for Chongqing Young and Middle-Aged Medical Excellence Team.

#### Author information

Shuai Yang

Department of Hematology & Oncology, Jiangbei Campus, The First Affiliated Hospital of Army Medical University, Chongqing 400038, China, Email: yang2008_342@foxmail.com

Lu Zheng

Department of Hepatobiliary Surgery, Xinqiao Hospital, Army Medical University, Chongqing 400037, China, Email: xqyyzl1@163.com

Lingling Li

State Key Laboratory of Genetic Engineering, Institutes of Biomedical Sciences, Human Phenome Institute, School of Life Sciences, Zhongshan Hospital, Fudan University, Shanghai 200433, China, Email: lill18@fudan.edu.cn

Jiangang Zhang

Department of Medical Oncology, Chongqing University Cancer Hospital, Chongqing 400030, China, Email: zhangjiangang25@163.com

Jingchun Wang

Department of Gastroenterology, Xinqiao Hospital, Army Medical University, Chongqing 400037, China, Email: 1065060238@qq.com

Huakan Zhao

Department of Medical Oncology, Chongqing University Cancer Hospital, Chongqing 400030, China, Email: ZHKK2011@126.com

Yu Chen

Department of Medical Oncology, Chongqing University Cancer Hospital, Chongqing 400030, China, Email: 466420109@qq.com

Xudong Liu

Department of Medical Oncology, Chongqing University Cancer Hospital, Chongqing 400030, China, Email: liuxubio@126.com

Hui Gan

Department of Radiology, Xinqiao Hospital, Army Medical University, Chongqing 400037, China, Email: ganhui200497@163.com

Junying Chen

Department of Pathology, Jiangbei Campus, The First Affiliated Hospital of Army Medical University, Chongqing 400038, China, Email: 1666549953@qq.com

Mei Yan

Department of Pathology, Jiangbei Campus, The First Affiliated Hospital of Army Medical University, Chongqing 400038, China, Email: 793507730@qq.com

Chuanyin He

Department of Pathology, Jiangbei Campus, The First Affiliated Hospital of Army Medical University, Chongqing 400038, China, Email: 782292022@qq.com

Kai Li

State Key Laboratory of Genetic Engineering, Institutes of Biomedical Sciences, Human Phenome Institute, School of Life Sciences, Zhongshan Hospital, Fudan University, Shanghai 200433, China, Email: 20110700097@fudan.edu.cn

Chen Ding

State Key Laboratory of Genetic Engineering, Institutes of Biomedical Sciences, Human Phenome Institute, School of Life Sciences, Zhongshan Hospital, Fudan University, Shanghai 200433, China, Email: chend@fudan.edu.cn

**Yongsheng Li (Correspondence)**

Department of Medical Oncology, Chongqing University Cancer Hospital, Chongqing 400030, China, Email: [lys@cqu.edu.cn](mailto:lys@cqu.edu.cn)

Shuai Yang, Lu Zheng and Lingling Li contributed equally to this work.

### Supplementary methods

#### Protein Extraction, Trypsin Digestion and Liquid Chromatography-tandem Mass Spectrometer (LC-MS/MS) Analysis

The samples underwent three washes in PBS to remove blood and debris. Then, the tissues were minced and suspended in lysis buffer (8 M urea, 100 mM Tris-hydrochloride, pH 8.0) before being sonicated for 1 minute (5 seconds on and 5 seconds off, amplitude 25%). The lysates were then centrifuged at 14,000 × g (precooled at 4 °C) for 10 minutes, and the resulting supernatant was collected as whole-tissue extracts. The protein concentration in the extracts was determined using the Bradford protein assay (TaKaRa, T9310A). Next, the extracts (100 μg) were reduced with 10 mM dithiothreitol at 56 °C for 30 min and alkylated with 10 mM iodoacetamide at room temperature for 30 min in the dark. Subsequently, the protein samples were digested with trypsin using the filter-aided sample preparation (FASP) method. The protein solution was loaded into a 30 kD Microcon filter tube (Sartorius) and centrifuged at 12,000 g for 20 minutes. The precipitate in the filter was washed twice by adding 200 μL of 50 mM NH_4_HCO3. The precipitate was then resuspended in 50 μL of 50 mM NH_4_HCO_3_. Protein samples were subjected to trypsin digestion using an enzyme-to-substrate ratio of 1:50 at 37 °C for 18-20 hours in the filter. The digested samples were collected by centrifugation at 12,000 g for 15 minutes. To enhance the yield, the collected samples were washed twice with 200 μL of MS water. The AQ model Vacuum concentrator from Eppendorf, Germany was used to pump out the centrifugate.

To profile the proteome, we utilized the Q Exactive HF-X tandem mass spectrometer (MS/MS) in conjunction with an Easy-nLC 1200 liquid chromatography (LC) system and a Q Exactive HF-X nano-electrospray ion source (Thermo Fisher Scientific) for peptide sample analysis. Dried peptide samples were dissolved in Solvent A, which consisted of 0.1% formic acid in water. The samples were loaded onto a 2-cm self-packed trap column with 100 μm inner diameter and 3 μm ReproSil-Pur C18-AQ beads from Dr Maisch GmbH, using Solvent A. Separation was carried out on a 150 μm inner diameter column with a length of 15 cm, packed with 1.9 μm ReproSil-Pur C18-AQ beads also from Dr Maisch GmbH. The separation was performed over a 75-minute gradient using Solvent A (0.1% formic acid in water) and Solvent B (0.1% formic acid in 80% ACN) at a constant flow rate of 600 nL/min (0-75 min, 0 min, 4% B; 0-10 min, 4-15% B; 10-60 min, 15-30% B; 60-69 min, 30-50% B; 69-70 min, 50-100% B; 70-75 min, 100% B). The eluted peptides were ionized at 2 kV and then subjected to MS analysis. The analysis was conducted using an Orbitrap mass analyzer with a mass resolution of 120,000. Full scans were performed in the m/z range of 300 – 1400. The top 60 precursor ions were selected for fragmentation in an HCD cell with a normalized collision energy of 27%. The resulting fragment ions were transferred to the Orbitrap analyzer, which operated at a resolution of 7500. The automatic gain control (AGC) was set to 3e6 for full MS and 5e4 for MS/MS. The maximum ion injection times were set to 80 ms. Dynamic exclusion of previously acquired precursor ions was enabled for 12 seconds. The data quality was assessed using internal standards and quality control samples. To ensure quality control, Spearman's correlation coefficient was computed for all runs using HEK293T cell samples.

#### Peptide Identification and Protein Quantification

LC-MS/MS generated MS raw files were analyzed using 'Firmiana', a comprehensive proteomic cloud platform (<https://phenomics.fudan.edu.cn/firmiana/>).^1^ The Mascot search engine was employed to compare the data against the human NCBI reference proteome database (updated on 04–07-2013). Trypsin/P was used as the protease with a maximum of two allowed missed cleavages. A mass tolerance of 10 ppm for precursor and 50 mmu for production was permitted. The fixed modification was carbamidomethyl (C), while acetylation (Protein N-term) and oxidation (M) were considered as variable modifications. The peptide cutoff for false discovery rate (FDR) was set at 1% using a target-decoy strategy. Each peptide was either assigned as a unique peptide to a specific protein group or set as a razor peptide to a single protein group with the highest peptide evidence. The protein groups assembled by 'Firmiana' were also filtered to 1% protein-level FDR using the target-decoy strategy.

#### Extraction of Lipids and Metabolites

After thawing the sample slowly at 4°C, 30 mg of tissue was transferred to a 1.5 mL centrifuge tube. A pre-cooled methanol/water solution (2:1) of 300 μL was added to the tube, followed by vortexing for 30 seconds to homogenize the tissue. Next, 600 μL of MTBE solution was added and the tube was vortexed again for 30 seconds. The tube was then cooled in an ice bath and subjected to ultrasonication for 10 minutes. After ultrasonication, the tube was centrifuged at 14,000 g and 4°C for 10 minutes, resulting in the separation of the solution into upper and lower layers. The upper layer, containing the lipids, was collected in a new EP tube, while the lower layer, containing the metabolites, was collected in a different EP tube. Finally, the samples were dried under vacuum at room temperature and stored at -80°C.

#### LC-MS/MS Analysis of Lipids

The mobile phase A and B are identical in both of negative and positive mode. Thereinto, mobile phase A was consisted of 10 mM ammonium formate, acetonitrile and water in a 60:40 ratio, along with 0.1% formic acid; mobile phase B was consisted of 10 mM ammonium formate, isopropyl alcohol and acetonitrile in a ratio of 50:50, along with 0.1% formic acid. The sample was separated using the microflow rate ultra-high performance liquid chromatography system Nexera UHPLC LC-30A. Initially, the chromatographic column was equilibrated with 98% of mobile phase A. Subsequently, the sample was delivered to the Lipid column (Thermo, Acclaim C30, 3μm, 2.1×100 mm column) using an autosampler, at a flow rate of 0.26 mL/min. The gradient elution was performed as follows: starting with 30% of mobile phase B, followed by a linear increase from 30% to 100% of mobile phase B over the next 20 minutes. After 5 minutes at 100% of mobile phase B, there was a 0.1-minute adjustment back to 30% of mobile phase B, followed by a 2.9-minute cleaning step.

After the separation on the chromatographic column, the samples were analyzed using the Q Exactive HF-X mass spectrometer. The detection was performed in both positive and negative ion modes. The full scan range for the parent ions was set from 200 to 2000 m/z. For MS1, the resolution was set to 120,000, with an Automatic Gain Control (AGC) target set to 1e6 and a maximum ionization time (Maximum IT) of 100 ms. For MS2, the resolution was set to 15,000, with an AGC target of 2e5 and a maximum ionization time of 80 ms. The fragmentation was carried out using the High-Energy Collision Dissociation (HCD) mode, with normalized collision energy values of 20, 40, and 60. The isolation window was set at 1.5 m/z.

#### Lipid Identification and Quantification

The raw data collected from the mass spectrometry analysis (RAW files) were processed using Progenesis QI software, a commercial lipidomics software platform developed by Waters, for database searching, the Lipid-MAPS was utilized as the target database. The search parameters included a parent tolerance of 5 ppm and a product tolerance of 5 ppm, ultimately obtaining identification information for the samples. We retained peak area data for which missing values did not exceed 50% within the experimental samples, and excluded peak area data points with a relative standard deviation (RSD) exceeding 30% within the quality control (QC) samples. Specifically, missing values were imputed using a method involving one-tenth of the minimum value for each respective feature. Finally, annotate the obtained high-quality features according to the LIPID MAPS database.

#### LC-MS/MS Analysis of Metabolites

The positive mode mobile phase A consists of 10 mM ammonium acetate, acetonitrile, and water in a ratio of 95:5, along with 0.1% formic acid. Conversely, the positive mode mobile phase B comprises 10 mM ammonium acetate, acetonitrile, and water in a ratio of 50:50, along with 0.1% formic acid. On the other hand, the negative mode mobile phase A consists of 10 mM ammonium acetate and a mixture of acetonitrile and water in a ratio of 95:5, with the pH adjusted to 8.0 using ammonia solution. Similarly, the negative mode mobile phase B consists of 10 mM ammonium acetate and a mixture of acetonitrile and water in a ratio of 50:50, with the pH adjusted to 8.0 using ammonia solution.

The sample was analyzed using the Nexera UHPLC LC-30A system. The chromatographic column was initially equilibrated with 98% mobile phase A. The sample was then delivered to the HILIC column (Waters, ACQUITY UPLC BEH Amide 1.7 μm, 2.1×100 mm column) via an autosampler at a flow rate of 0.3 mL/min. The gradient elution process involved starting with 2% mobile phase B and maintaining it for 0.5 minutes. This was followed by a linear increase from 2% to 98% mobile phase B over the next 11.5 minutes. After maintaining 98% mobile phase B for 4 minutes, there was a brief adjustment back to 2% mobile phase B for 0.1 minutes. This was then followed by a 1.9-minute cleaning step.

Following separation on the chromatographic column, the samples were analyzed using the Q Exactive HF-X mass spectrometer in both positive and negative ion modes. The parent ions were scanned within a range of 70 to 1050 m/z. In the first stage of mass spectrometry (MS1), the resolution was set to 120,000. An Automatic Gain Control (AGC) target of 3e6 was also set, along with a maximum ionization time of 100 ms. In the second stage (MS2), the resolution was set to 7,500, with an AGC target of 2e5 and a maximum ionization time of 50 ms. The High-Energy Collision Dissociation (HCD) mode was used to fragment the sample, with normalized collision energy values of 20, 40, and 60. The isolation window was set at 1.5 m/z, and the daughter ions were scanned in the range of 200 to 2000 m/z.

#### Metabolite Identification and Quantification

For metabolite identification and quantification, the MS raw files were analyzed by Compound Discoverer (Thermo Fisher Scientific, CA, USA). The chemical standards and manually curated compound list was identified by our in-house metabolite library of mzCloud and mzVault databases based on accurate mass (m/z, ± 5 ppm), retention time and spectral patterns. Next, the identified compounds were also searched against public databases, including human metabolome database (HMDB), PubChem Compound database, Chemical Entities of Biological Interest database and Kyoto Encyclopedia of Genes and Genomes (KEGG) compound database for further annotation.

Reference

1. Feng, J.W., Ding, C., Qiu, N.Q.*, et al*. Firmiana: towards a one-stop proteomic cloud platform for data processing and analysis. *Nature Biotechnology*. 2017;35:409-412. doi:10.1038/nbt.3825.
